# E6AP Promotes a Metastatic Phenotype in Prostate Cancer

**DOI:** 10.1016/j.isci.2019.10.065

**Published:** 2019-11-02

**Authors:** Cristina Gamell, Ivona Bandilovska, Twishi Gulati, Arielle Kogan, Syer Choon Lim, Zaklina Kovacevic, Elena A. Takano, Clelia Timpone, Arjelle D. Agupitan, Cassandra Litchfield, Giovanni Blandino, Lisa G. Horvath, Stephen B. Fox, Scott G. Williams, Andrea Russo, Enzo Gallo, Piotr J. Paul, Catherine Mitchell, Shahneen Sandhu, Simon P. Keam, Sue Haupt, Des R. Richardson, Ygal Haupt

**Affiliations:** 1 Tumour Suppression Laboratory, Peter MacCallum Cancer Centre, 305 Grattan St, Melbourne, VIC 3000, Australia; 2 Sir Peter MacCallum Department of Oncology, The University of Melbourne, Parkville, VIC 3010, Australia; 3 Department of Pathology and Bosch Institute, University of Sydney, Sydney, NSW 2006, Australia; 4 Department of Pathology, Peter MacCallum Cancer Centre, Melbourne 3000, Australia; 5 IRCCS Regina Elena National Cancer Institute, Rome, Italy; 6 The Chris O'Brien Lifehouse, Sydney, NSW 2050, Australia; 7 Garvan Institute of Medical Research, Sydney, NSW 2010, Australia; 8 Division of Radiation Oncology and Cancer Imaging, Peter MacCallum Cancer Centre, Melbourne, VIC 3000, Australia; 9 Department of Pathology and Biological Responses, Nagoya University Graduate School of Medicine, Nagoya 466-8550, Japan; 10 Department of Clinical Pathology, University of Melbourne, Parkville, VIC 3010, Australia; 11 Department of Biochemistry and Molecular Biology, Monash University, Melbourne 3800, Australia

**Keywords:** Biological Sciences, Cell Biology, Cancer

## Abstract

Although primary prostate cancer is largely curable, progression to metastatic disease is associated with very poor prognosis. E6AP is an E3 ubiquitin ligase and a transcriptional co-factor involved in normal prostate development. E6AP drives prostate cancer when overexpressed. Our study exposed a role for E6AP in the promotion of metastatic phenotype in prostate cells. We revealed that elevated levels of E6AP in primary prostate cancer correlate with regional metastasis and demonstrated that E6AP promotes acquisition of mesenchymal features, migration potential, and ability for anchorage-independent growth. We identified the metastasis suppressor NDRG1 as a target of E6AP and showed it is key in E6AP induction of mesenchymal phenotype. We showed that treatment of prostate cancer cells with pharmacological agents upregulated NDRG1 expression suppressed E6AP-induced cell migration. We propose that the E6AP-NDRG1 axis is an attractive therapeutic target for the treatment of E6AP-driven metastatic prostate cancer.

## Highlights

• Elevated E6AP levels in primary PC in men correlate with regional metastasis
• Elevated E6AP levels promote mesenchymal features and migration potential
• E6AP promotes a metastatic phenotype by reducing NDRG1 expression levels
• Pharmacological upregulation of NDRG1 suppresses E6AP-induced cell migration

## Graphical Abstract

**Figure:**
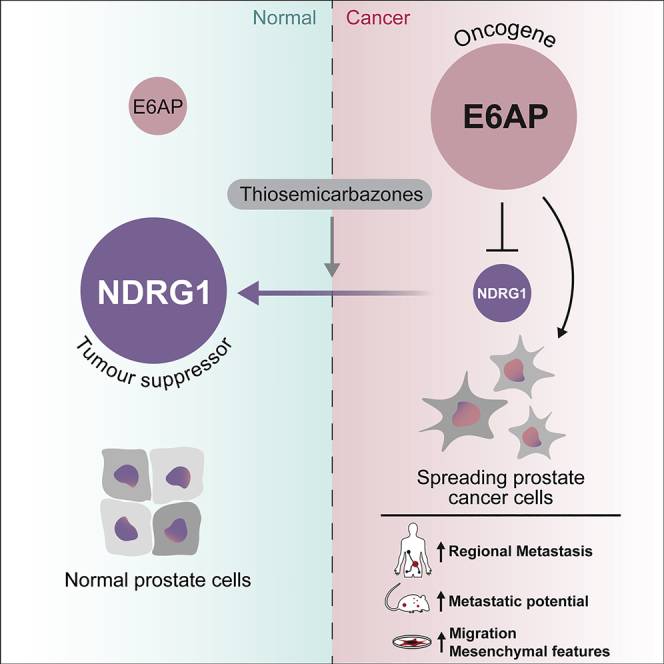

## Introduction

Metastatic prostate cancer (PC) is a major health problem that results in death in two-thirds of its patients within five years (Siegel et al., 2018). Although the majority of prostate cancer patients have an initial response to androgen-deprivation therapy, most men with metastases develop resistance to primary hormone therapies: a condition termed metastatic castration-resistant PC. A detailed understanding of the molecular drivers of progression to metastatic disease is therefore urgently needed to design rational therapeutic strategies to improve patient outcomes.

Previous studies by others and us support a role for E6AP in driving PC (Birch et al., 2014, Paul et al., 2016, Raghu et al., 2017). The human papilloma virus (HPV) E6-associated protein (E6AP) was originally identified as the E3 ligase that is recruited by the E6 protein of HPV to promote p53 for proteasomal degradation (31). In addition to its E3 ligase activity, E6AP can also act as a transcription co-factor, as demonstrated by its role in regulating the transcriptional activities of nuclear hormone receptors (Nawaz et al., 1999, Raghu et al., 2017) and of E2F-1, as we recently showed (Gamell et al., 2017a, Raghu et al., 2017).

Beyond the context of HPV-related cancer, several lines of evidence strongly support a role for E6AP in the pathogenesis of PC. First, E6AP is required for normal prostatic development since its deficiency results in a 40% reduction in the size of the prostate gland (Khan et al., 2006). Second, over-expression of E6AP in the prostate gland increases proliferation and drives prostate intraepithelial neoplasia (PIN) (Khan et al., 2006, Srinivasan and Nawaz, 2011). Third, a partial loss of E6AP expression is sufficient to attenuate PC cell growth *in vitro* and *in vivo* (Paul et al., 2016). These effects of E6AP on prostate cells are mediated, at least in part, through the downregulation of the key tumor suppressors: PML (Paul et al., 2016), p27Kip1 (Raghu et al., 2017), and clusterin (Gulati et al., 2018). High levels of E6AP in localized PC correspond with elevated Gleason scores and poor patient prognosis (Birch et al., 2014). The latter study also indicated that primary human prostate tumors with high levels of E6AP tend to be associated with increased rates of distant metastasis (Birch et al., 2014). This encouraged us to explore the role of E6AP in metastatic PC in the present study.

Several tumor suppressors, such as PTEN, are known to suppress metastatic cancer (Bandyopadhyay et al., 2004). Among these, N-myc downstream regulated gene 1 (NDRG1) has been shown to act as a tumor and metastasis suppressor gene in multiple cancers, including PC (Bandyopadhyay et al., 2003, Chung et al., 2012, Song et al., 2010). Over-expression of NDRG1 in PC cells suppressed the metastatic phenotype *in vitro* and *in vivo* (Bandyopadhyay et al., 2003, Sharma et al., 2017, Sun et al., 2013), and its expression correlates with lower disease progression (Sun et al., 2013). Further, over-expression of NDRG1 downregulates key signaling proteins and pathways, including those mediated by TGFβ (Dixon et al., 2013, Kovacevic et al., 2013), WNT (Jin et al., 2014), and PI3K/AKT (Kovacevic et al., 2013). Furthermore, NDRG1 has been demonstrated to upregulate the tumor suppressors PTEN and SMAD4 (Kovacevic et al., 2013). The expression of NDRG1 in PC cells is induced under a wide variety of stress and cell-growth regulatory conditions. These include responses to hypoxic stress, which is HIF1-α-dependent (Cangul, 2004, Le and Richardson, 2004), and to androgen levels (Ulrix et al., 1999). NDRG1 is largely regulated at the protein level by a variety of mechanisms.

Given its role in the suppression of metastatic cancer, elevating NDRG1 levels has been considered as a promising therapy (Le and Richardson, 2004). A potential strategy for empowering NDRG1 is through exposure to a unique class of thiosemicarbazones (Whitnall et al., 2006, Yuan et al., 2004). Specific application to cancer prompted the design of agents known as di-2-pyridylketone thiosemicarbazones. These include di-2-pyridylketone 4-cyclohexyl-4-methyl-3-thiosemicarbazone (DpC), which has advanced to clinical trials, and the model compound di-2-pyridylketone 4,4-dimethyl-3-thiosemicarbazone (Dp44mT). These agents demonstrate potent and selective anti-tumour activity *in vitro* and *in vivo* (reviewed in Guo et al., 2016, Jansson et al., 2015; and Xu et al., 2018) Further, these agents inhibit metastasis *in vivo*, in a manner that depends on NDRG1 expression (Li et al., 2016, Liu et al., 2012).

This study builds on our recent transcriptomic and proteomic screens (Gulati et al., 2018), which identified a link between E6AP and metastatic processes in PC cells. We demonstrate that high levels of E6AP in primary PC are often associated with loco-regional metastasis. We show that E6AP promotes mesenchymal characteristics that are enhanced by treatment with TGFβ and are associated with the upregulation of associated markers, including *SNAI2* (Slug) and *SNAI1* (Snail). This is consistent with the promotion of metastasis *in vivo* by E6AP. By mining our screen, we identified NDRG1 as a new downstream target of E6AP. Compounds that activate NDRG1 counter these cancer-promoting E6AP phenotypes. Overall, we conclude that E6AP is a key driver of PC metastasis, rendering the E6AP-NDRG1 axis an attractive therapeutic target for metastatic PC.

## Results

### Elevated Levels of E6AP in Primary PC Samples Are Associated with Regional Metastasis

We previously demonstrated that PC patients expressing high levels of E6AP protein in their primary tumor have increased rates of distant metastasis as compared with patients with low levels of E6AP (Birch et al., 2014). On this basis, we hypothesized that E6AP plays a role in the promotion of metastasis.

**Figure 1:**
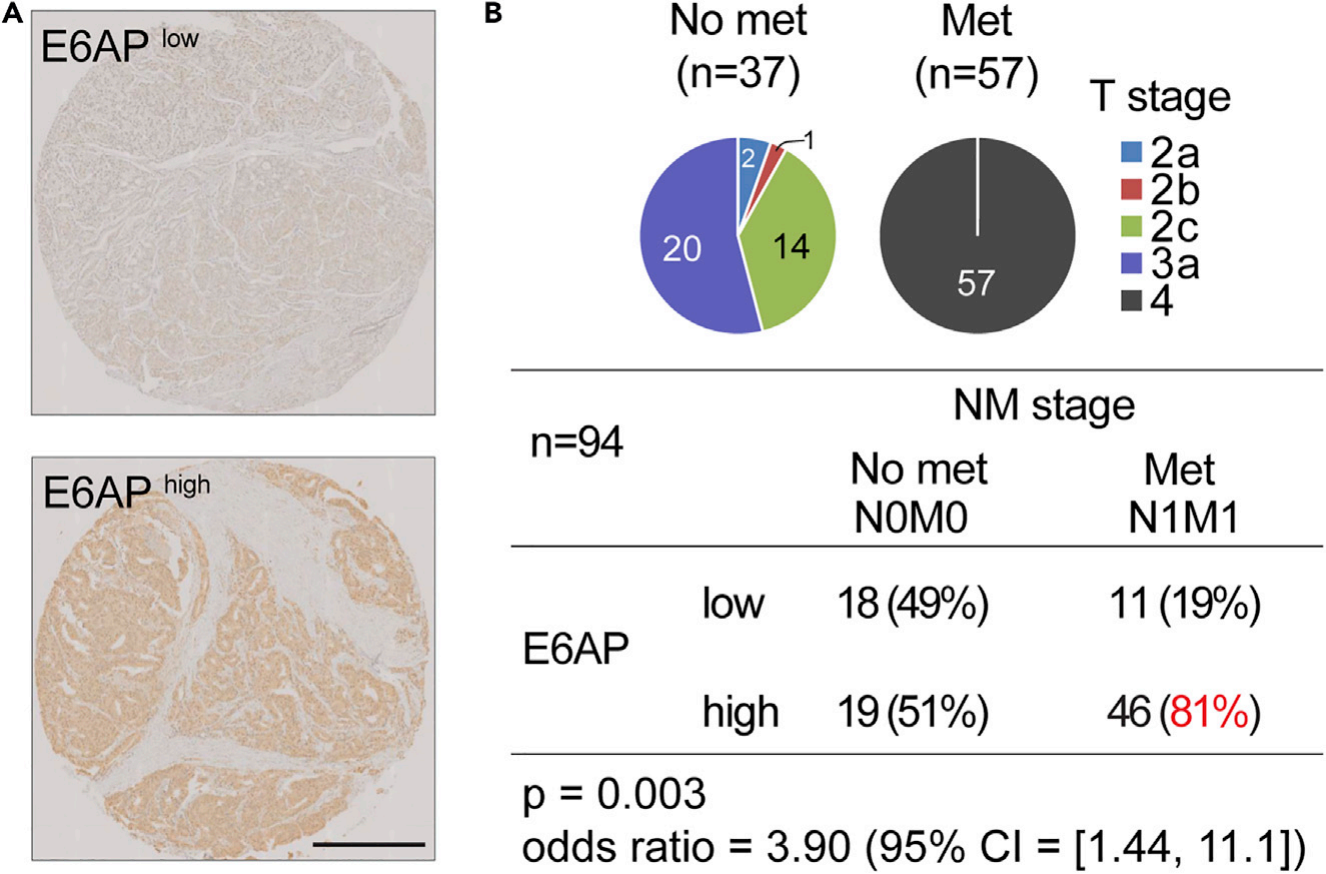
High E6AP Expression Levels Predict Early Metastasis Following Prostatectomy (A) Representative IHC images of E6AP staining in prostate tumors. Scale bar represents 500μm. (B) Pie charts indicate T stage distribution and number of patients in each cohort. All patients in Met category were node-positive (i.e. Stage IV). The numbers inside the brackets indicate the number of patients in each group. Staining intensities for E6AP were calculated for patients who did not present metastasis in the regional lymph nodes when undergone prostatectomy (“No met”) and those who did (“Met”).

To test this hypothesis, we measured E6AP protein levels by immunostaining tissue microarrays (TMA, refer to Methods) of primary biopsies from PC patients who underwent radical prostatectomy (*n* = 94) (Figure 1). Among these, more than half presented with loco-regional lymph node metastasis at the time of surgery (*n* = 57). The great majority (81%) of lymph node positive patients expressed high levels of E6AP (Figure 1B). Therefore, these correlative clinical findings suggest a role for E6AP in the promotion of metastasis and raise the possibility that E6AP may serve as a predictive biomarker for aggressive PC phenotype.

### E6AP Knockdown Is Associated with Loss of Mesenchymal Features

**Figure 2:**
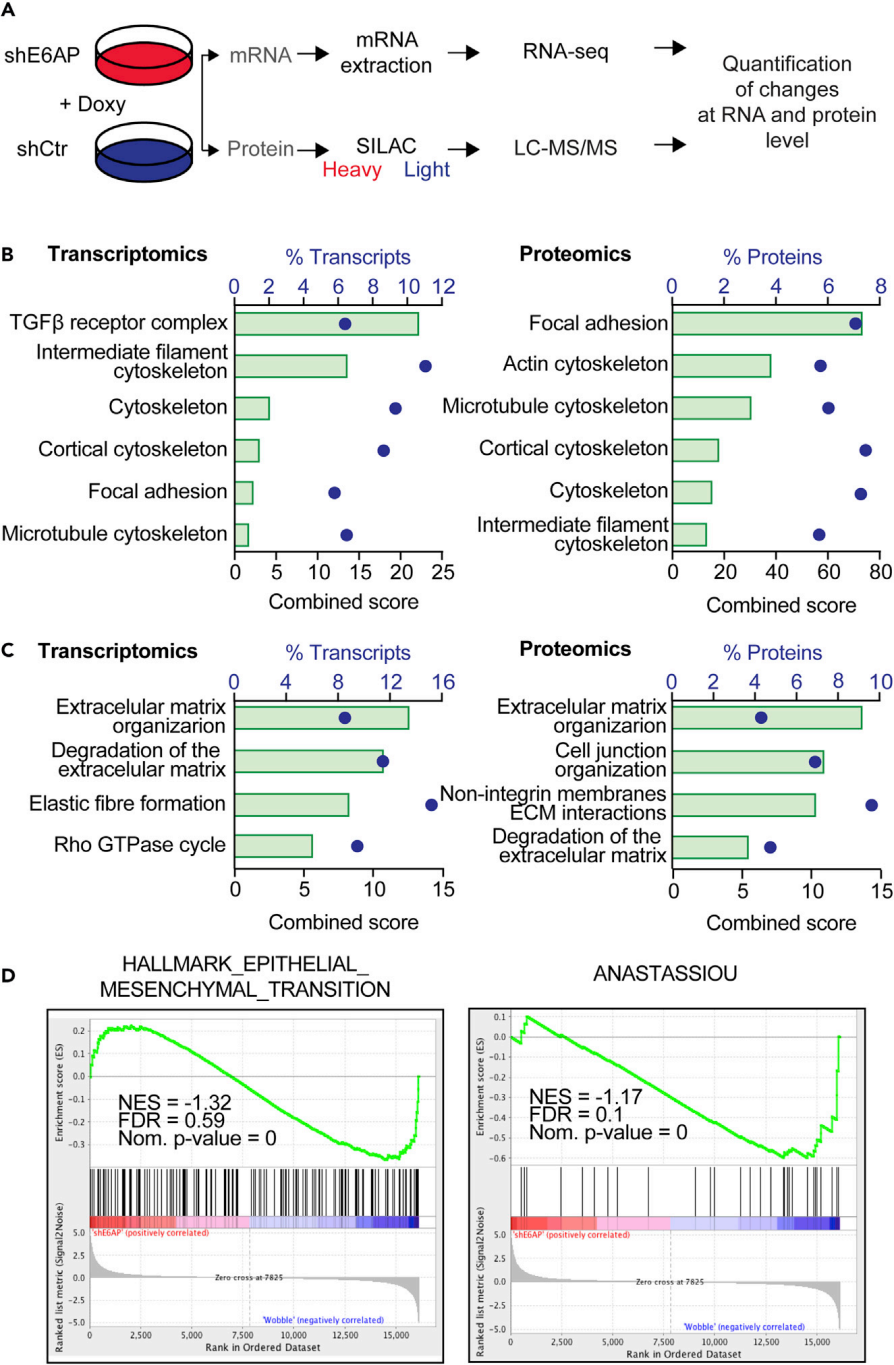
Loss of E6AP Leads to a Decrease Mesenchymal Genetic and Proteomic Program (A–C) (A) Schematic representation of the transcriptomic (RNAseq) and proteomic (SILAC LC-MS/MS) screens to identify targets of E6AP (Gulati et al., 2018). Functional gene ontology analysis of E6AP signatures in transcriptomics and proteomics data using either (B) GO cellular components or (C) Reactome pathway databases. Significant pathways enriched upon E6AP KD are presented as combined score (green bar) and percentage of transcripts (blue dots) associated with significantly altered transcripts (left) and proteins (right). (D) GSEA enrichment score plots from RNA-Seq showing inverse correlation between E6AP expression levels and genes regulating EMT (hallmark EMT signature and the published Anastassiou EMT gene signature (Anastassiou et al., 2011)). NES, normalized enrichment score; FDR, false-discovery rate.

To assess the potential role of E6AP in the promotion of metastasis, we analyzed changes in global expression profiles that occur upon knockdown (KD) of E6AP (Gulati et al., 2018). For this purpose, we studied the output from our recent discovery screens, combining transcriptomic (RNAseq) and proteomic (LC-MS/MS SILAC-labeled) data. We compared the expression profiles of the metastatic PC cell line, DU145, transduced with a doxycycline (Doxy)-inducible short hairpin RNA (shRNA) to KD E6AP (shE6AP) versus a wobble shRNA control (shCtr; Figure 2A) (Gulati et al., 2018).

We performed gene ontology on differentially expressed genes (RNAseq) and proteins (proteomics) upon E6AP KD. This analysis revealed enrichment of cellular components that are consistent with cell reprogramming to favor the loss of the mesenchymal phenotype (Figure 2B). Notably, during cancer progression, it is the adoption of mesenchymal properties by epithelia that promote invasion, and intra- and extravasation, which defines a key step underpinning metastasis (Thiery, 2002). Significant changes pertained to the cortical cytoskeleton, focal adhesion, actin cytoskeleton, and microtubule cytoskeleton (Figure 2B). We also assessed the enriched biological pathways associated with E6AP KD by interrogating the Reactome database. Consistent with the potential for E6AP to promote mesenchymal phenotypes, biological pathways linked to extracellular matrix organization and cell morphology were significantly (p < 0.05) enriched at the transcriptional and protein levels (Figure 2C).

To extend these findings beyond gene ontology and pathway analyses, we performed gene set enrichment analysis (GSEA) on the RNAseq data, using all the quantified genes. GSEA was used to compare between E6AP KD and control cells at the level of gene sets, using gene expression profiles, rather than the significantly dysregulated genes. Consistent with the gene ontology and pathway analyses, GSEA analysis indicated that in response to E6AP KD, there was a negative enrichment of signatures involved in mesenchymal transition (hallmark epithelial-mesenchymal transition [EMT] and Anastassiou mesenchymal transition signature) (Figure 2D). Of note, GSEA was not performed with the proteomics data because GSEA compares two conditions, and in our SILAC-labeling process the two groups were mixed 1:1 ratio prior to digestion with trypsin.

These analyses strongly suggest that E6AP induces expression profile changes that are consistent with mesenchymal phenotype, which supports a role for E6AP in the promotion of metastasis of PC.

### E6AP Knockdown Reduces the Metastatic Potential of PC Cells

**Figure 3:**
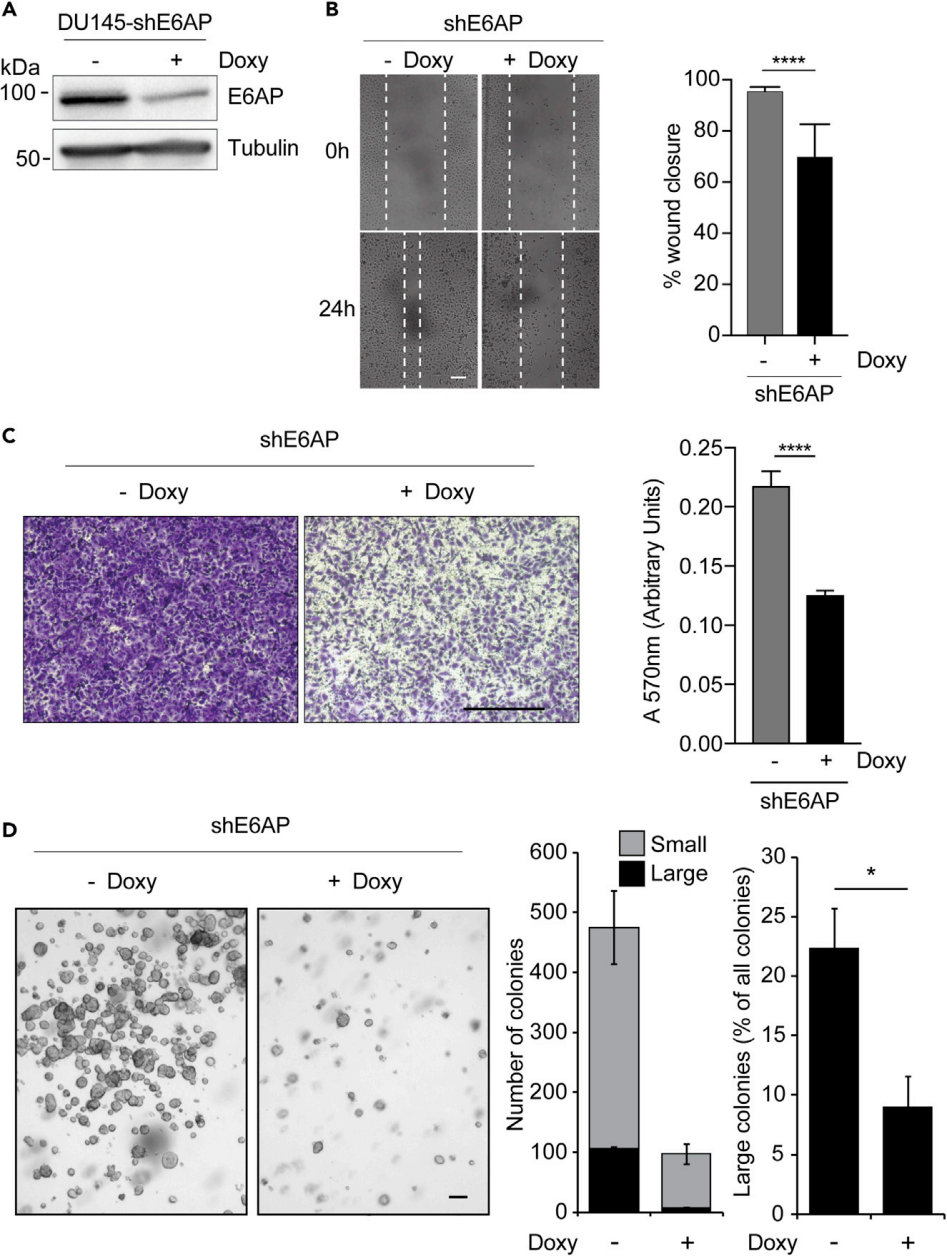
E6AP Knockdown Reduces the Metastatic Potential of PC Cells *In Vitro* (A) Immunoblot confirming KD of E6AP upon Doxy treatment for three days in DU145-shE6AP cells. (B and C) (B) DU145-shE6AP cells were treated with Doxy for three days before the confluent cell monolayer was scratched. Cells were then allowed to migrate in the presence or absence of Doxy for 24 h. Representative images are shown on the left panels and quantification of wound closure was calculated from triplicates and was obtained from multiple photographed fields taken immediately following the scratch and at the endpoint (right panel). Quantification of a representative experiment is shown as mean ± SD. ****p < 0.0001, unpaired *t* test. Scale bar represents 100 μm. (C) DU145-shE6AP cells were treated with Doxy for 2.5 days, seeded in transwells, and allowed to migrate in the presence or absence of Doxy for 24 h. Invaded cells were stained with crystal violet (left) and quantified at A_570_ after extraction of the dye (right). Each experiment was performed in duplicates and repeated three independent times. Quantification of a representative experiment is shown as mean ± SD. Scale bar represents 500μm. (D) DU145-shE6AP cells were grown in soft agar in the presence or absence of Doxy for 11 days. Fresh Doxy was added every three days. Representative images at experimental endpoint are shown. Quantification was based on duplicate experiments and unbiased computational detection and enumeration of colony size into small and large sizes using imaging software (large: >1,000 pixels, small 10–1,000 pixels). Large-size colony percentages were calculated as a proportion of total cells and/or colonies in each magnification field. Scale bar represents 100μm. ****p < 0.0001, unpaired *t* test.

To directly test the functional role of E6AP in the metastatic process, we examined whether KD of E6AP could reverse the highly metastatic mesenchymal-like DU145 PC cell line back to a more epithelial-like state. To measure the impact on migratory properties, we used the wound healing assay and the transwell migration assay. With both assays, KD of E6AP significantly (p < 0.0001) decreased the migratory capacity of the cells (Figures 3B, 3C, and S3). Notably, this effect was independent of any potential impact of E6AP downregulation on cell proliferation, because this decrease in cell migration remained in the presence of mitomycin C (4μg/mL), an inhibitor of DNA synthesis (Figures S1A and S1B). This effect was not specific for DU145, as similar results were obtained with another highly metastatic PC cell line, PC3 (Figures S1C and S1D).

Another characteristic of metastatic cells is their acquired ability for anchorage-independent growth. We assessed the effect of E6AP on this growth characteristic using a soft agar colony formation assay. In addition to reducing the number of overall cell numbers, Doxy-induced E6AP KD significantly (p < 0.05, n = 2) reduced the proportion of large colonies (Figure 3D). This supports a role for E6AP in promoting anchorage-independent growth.

**Figure 4:**
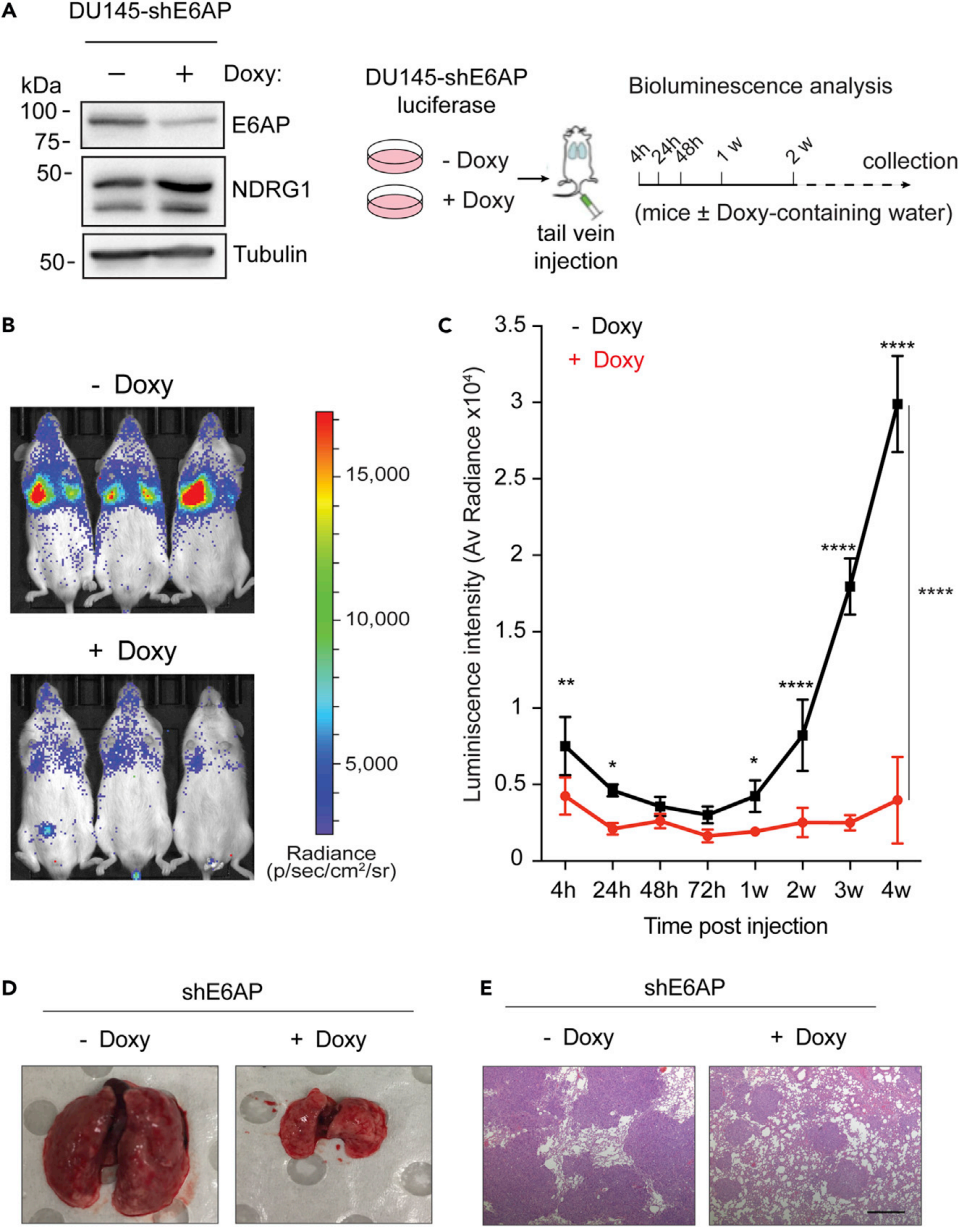
E6AP Knockdown Reduces the Metastatic Potential of PC Cells *In Vivo* (A) Western blot confirmation of E6AP KD and NDRG1 response in DU145 cells and schematic experimental plan of the metastasis of the tail vein experimental assay. DU145-shE6AP-luciferase cells were treated with Doxy for 2.5 days and injected in the tail vein of NSG mice (*n* = 6/group) maintained in Doxy-containing water. Control mice were not exposed to Doxy. Tumor burden was monitored 4 h, 24 h, and 48 h after injection of cells and then weekly *via* bioluminescence detection. (B) Representative bioluminescence images of animals in each experimental group one week after injection. (C) Quantification of the bioluminescence signal over the first 4 weeks (*n* = 6/group). Data are mean ± SEM. One-way and two-way ANOVA ****p < 0.0001, **p < 0.001, *p < 0.05. (D) Representative gross images of lung metastatic nodules from animals in each experimental group collected at endpoint. (E) Representative images of histologic sections stained with hematoxylin and eosin of lung metastasis from mice in each experimental group at endpoint collection. The highly densely stained areas represent metastatic nodules. Scale bar represents 500μm.

We next investigated whether KD of E6AP suppresses metastasis *in vivo*. For this purpose, we measured lung colonization and growth of DU145-shE6AP cells, pre-treated with Doxy for 2.5 days (to induce the shRNA expression) prior to tail vein injection into female NSG (NOD SCID gamma) mice (Figure 4A). Bioluminescence imaging of the luciferase-labeled cells revealed a significant (p < 0.0001) reduction in lung metastasis associated with E6AP KD after four weeks. This effect was already noticeable as early as 4 h post-injection, suggesting that KD of E6AP negatively affects the extravasation and/or early seeding of lung metastasis (Figures 4B and 4C). Continued monitoring over four weeks revealed a further reduction in lung metastatic outgrowth in mice injected with E6AP KD cells (Figures 4B and 4C). To confirm the bioluminescence measurements, lungs were analyzed at the time of autopsy anatomically and histologically, as measures of tumor burden. This significant difference was confirmed by a decrease in the number of metastatic lesions produced by DU145-shE6AP in the presence of Doxy as compared with mice maintained without Doxy (Figures 4D and 4E). Taken together, these analyses show that E6AP KD strongly inhibits PC metastasis *in vitro* and *in vivo*.

### Overexpression of E6AP Promotes Mesenchymal Characteristics of Prostate Epithelial Cells

**Figure 5:**
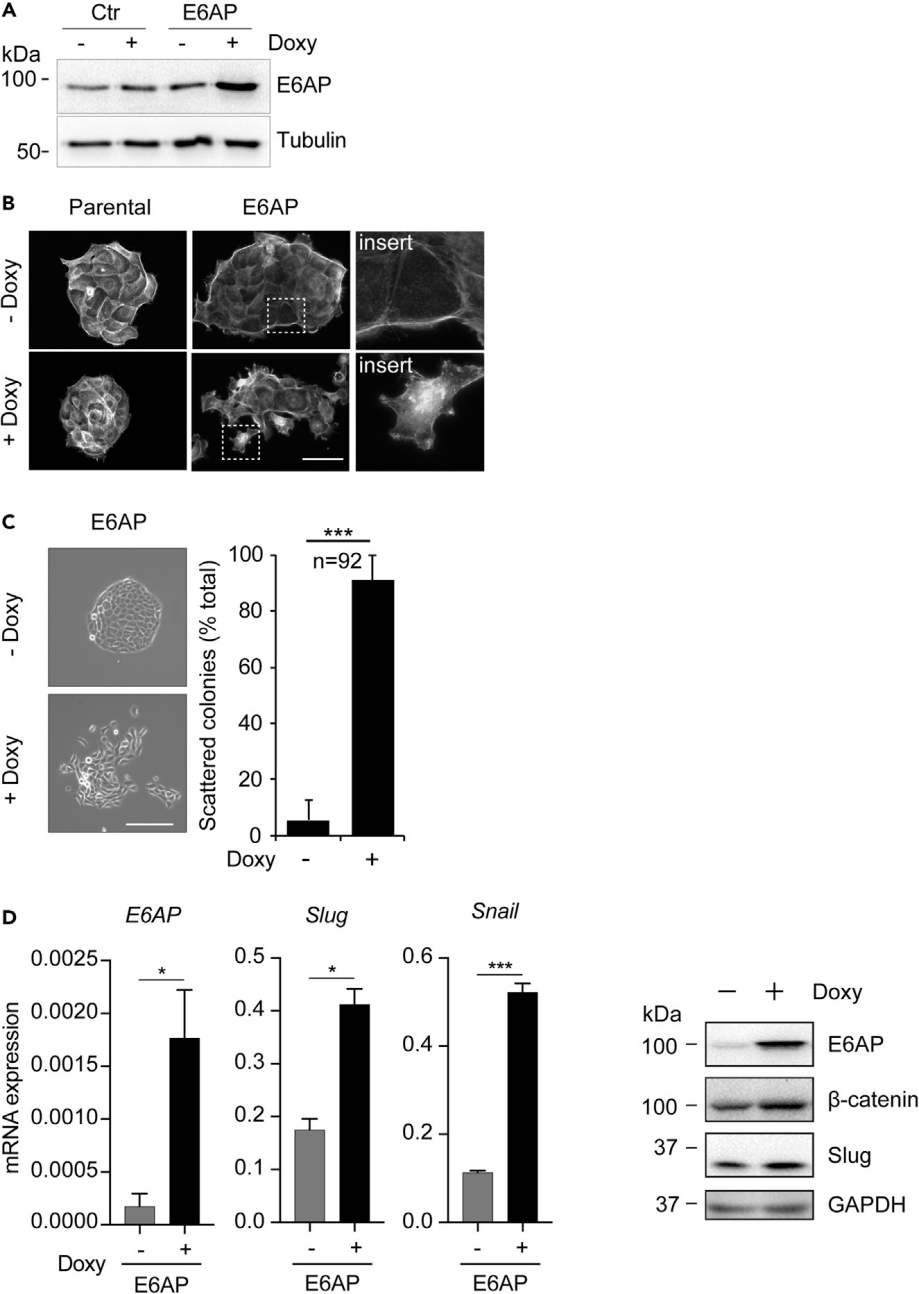
E6AP Overexpression in Epithelial-like BPH Cells Promotes Cell Morphology Changes Consistent with an Increased Metastatic Phenotype (A) Immunoblot confirming overexpression of E6AP upon Doxy treatment for three days in BPH-E6AP cells. (B) BPH-E6AP cells or the parental control cells were cultured for 3 days in the presence or absence of Doxy. Actin was then visualized with rhodamine-conjugated phalloidin using a fluorescent microscope. Scale bar represents 50μm. Each experiment was performed in at least triplicates and repeated three independent times. (C) BPH-E6AP cells or the parental control cells were plated at low density and allowed to form colonies for five days in the presence or absence of Doxy. Representative phase-contrast images of the colonies formed are shown. Scale bar represents 200μm. Each experiment was performed in quadruplicate with a representative image used for enumeration based on colony morphology classification as either tight or scattered. Quantification of a total count of 92 colonies is shown. (D) BPH-E6AP cells exposed to Doxy for three days for RNA analysis and five days for protein analysis. RT-PCR analysis of the expression of *E6AP*, *Slug* and *Snail* mRNA is shown. Data are ΔΔC_t_±SD of technical triplicates of one of three independent experiments. *p < 0.05, ***p < 0.001, unpaired *t* test. Protein levels of Slug, β-Catenin, and E6AP have been determined by immunoblot, and GAPDH has been used as loading control.

To explore the levels at which E6AP regulates metastasis, we measured the effect of E6AP overexpression on prostate cell morphology in BPH, prostate hyperplasia cells that constitute an epithelial-like cell line that originated from a benign prostate hyperplasia. BPH cells were transduced with a Doxy-inducible plasmid to overexpress E6AP (Figure 5A, refer to Methods). E6AP overexpression dramatically altered BPH cells morphology, converting these from a cobblestone-like epithelial to a fibroblastic morphology, showing classical mesenchymal structural features such as extension of lamellipodia and filopodia (Figure 5B). Notably, Doxy treatment alone did not affect the morphology of the control cells, supporting the observed change to be mediated by E6AP expression.

To further support the above finding, we measured the effect of E6AP overexpression in a colony-scattering assay. Colony scattering is characterized by the loss of epithelial cell-cell junctions and the acquisition of motility, which can be quantified as a measure of the acquisition of a mesenchymal phenotype. The BPH parental and the BPH-E6AP cells were seeded at very low density and were grown for several days in the presence or absence of Doxy. Cells overexpressing E6AP formed significantly (p < 0.001) more scattered colonies (Figure 5C), whereas the control parental cells grew as compact colonies and displayed the tight cell-cell adherence characteristic of epithelial cells (Figure S1E). Similar results were observed in DU145 cells transduced with Doxy-induced overexpression of E6AP (Figure S2B). Consistent with these findings, overexpression of E6AP induced a significant (p < 0.001–0.05) increase in the mRNA expression of key mesenchymal markers, *Slug* and *Snail*, as compared with control cells (Figure 5D), as well as protein level of Slug and β-catenin (Figure 5D), and vimentin (Figure S5). Together, these results support a functional role for E6AP in promoting a mesenchymal-like phenotype in prostate cells.

### E6AP Modulates TGFβ-induced Mesenchymal Transition

TGFβ signaling has been shown to induce epithelial-to-mesenchymal transition in PC cells (Chen et al., 2012) and therefore is relevant to various stages of tumor progression in the context of PC, among other cancer types (Oft et al., 1998). The impact of E6AP expression on mesenchymal characteristics led us to determine whether E6AP is involved in TGFβ-mediated mesenchymal transition.

**Figure 6:**
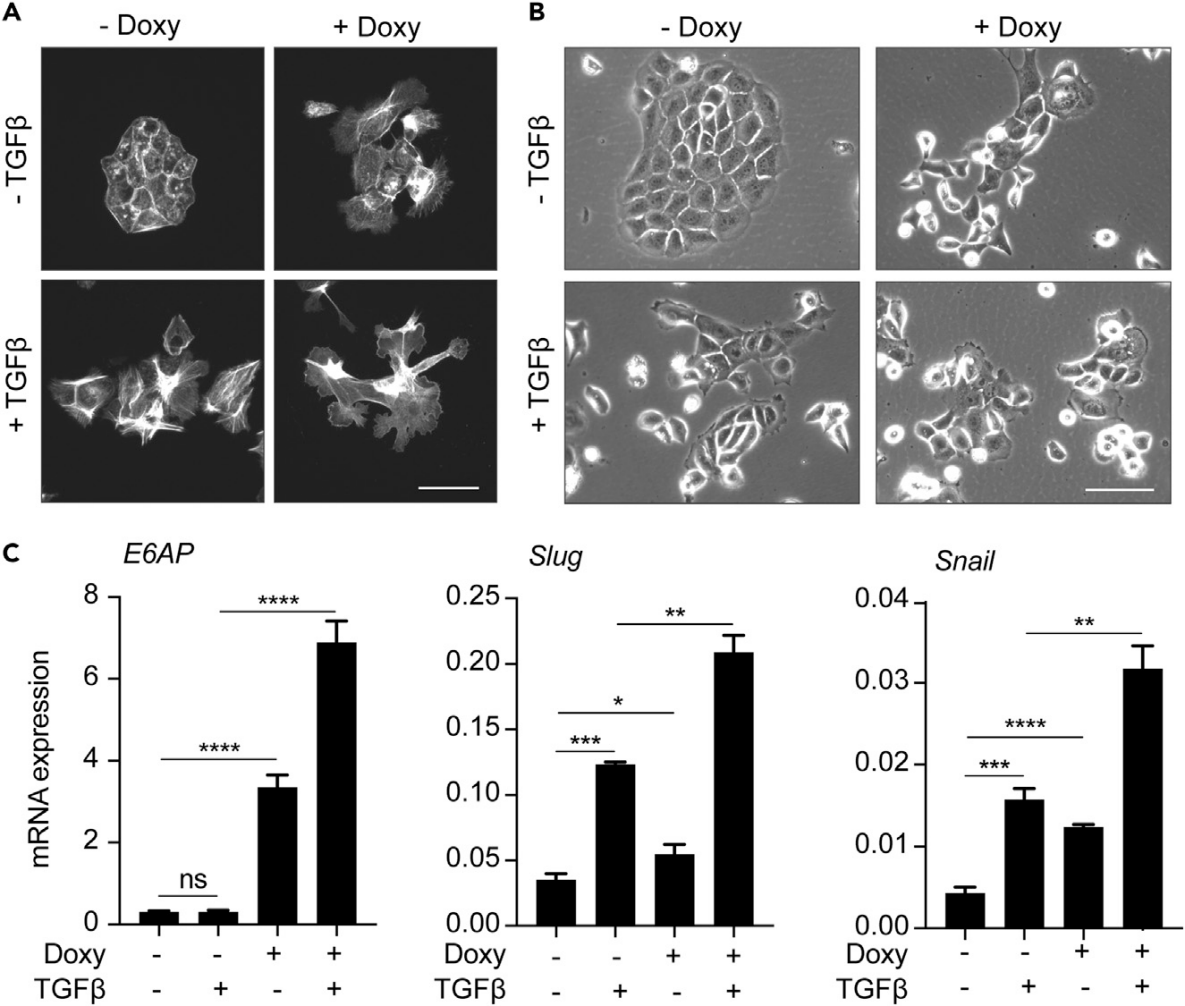
E6AP Enhances TGFβ-induced Mesenchymal Phenotype in BPH Cells (A) BPH-E6AP or the parental control cells were cultured for two days in the presence or absence of Doxy, serum-starved overnight and then stimulated with 10ng/mL TGFβ in media containing 0.05% FBS for a further two days in the presence or absence of Doxy. Actin was then visualized with rhodamine-conjugated phalloidin using a fluorescent microscope. Representative images are shown. Scale bar represents 50μm. Each experiment was performed in triplicates and repeated three independent times. A total of 10 colonies were counted per condition. (B) BPH-E6AP cells or the parental controls were plated at low density and allowed to form colonies for two days in the presence or absence of Doxy, serum-starved overnight and then stimulated with 10ng/mL TGFβ in media with 0.05% FBS for an additional two days in the presence or absence of Doxy. Representative phase-contrast images of the colonies formed are shown. Scale bar represents 100μm. Each experiment was performed in triplicates and repeated two independent times. (C) RT-PCR analysis of the expression of *E6AP*, *Slug* and *Snail* mRNA in BPH-E6AP cells grown for two days in the presence or absence of Doxy, serum-starved overnight and then stimulated with 10ng/mL TGFβ in media with 0.05% FBS for an extra two days (in the presence or absence of Doxy). Data are ΔΔC_t_±SD of technical triplicates of one of three independent experiments. *p < 0.05, **p < 0.01, ***p < 0.001, ****p < 0.0001, unpaired *t* test.

To determine whether TGFβ induces a mesenchymal phenotype in BPH prostate hyperplasia cells, we analyzed the effect of TGFβ on their cell morphology. Treatment of BPH cells with TGFβ resulted in marked morphological changes, notably the loss of a normal cuboidal epithelial shape and the acquisition of a more mesenchymal phenotype, characterized by dynamic protrusions (Figure 6A) and growth as scattered colonies (Figure 6B) lacking cell-cell contacts. Importantly, the acquisition of this TGFβ-induced mesenchymal phenotype was enhanced by E6AP overexpression, leading to an enhanced display of membrane protrusions (Figure 6A) and greater prevalence of a scattered growth pattern (Figure 6B). To explore the molecular changes associated with this transition, we measured the effect of TGFβ on E6AP-mediated induction of *Slug* and *Snail*. As expected, TGFβ treatment significantly (p < 0.0001–<0.01) upregulated the expression of these mesenchymal markers (Figure 6C), as did overexpression of E6AP, consistent with Figure 5D. Importantly, when E6AP-overexpressing cells were incubated with TGFβ, we observed a further increase in *Slug* and *Snail* expression (Figure 6C). These studies indicate that E6AP overexpression further potentiates a TGFβ-induced mesenchymal switch.

### E6AP Reduces the Levels of NDRG1 in PC Cell Lines

**Figure 7:**
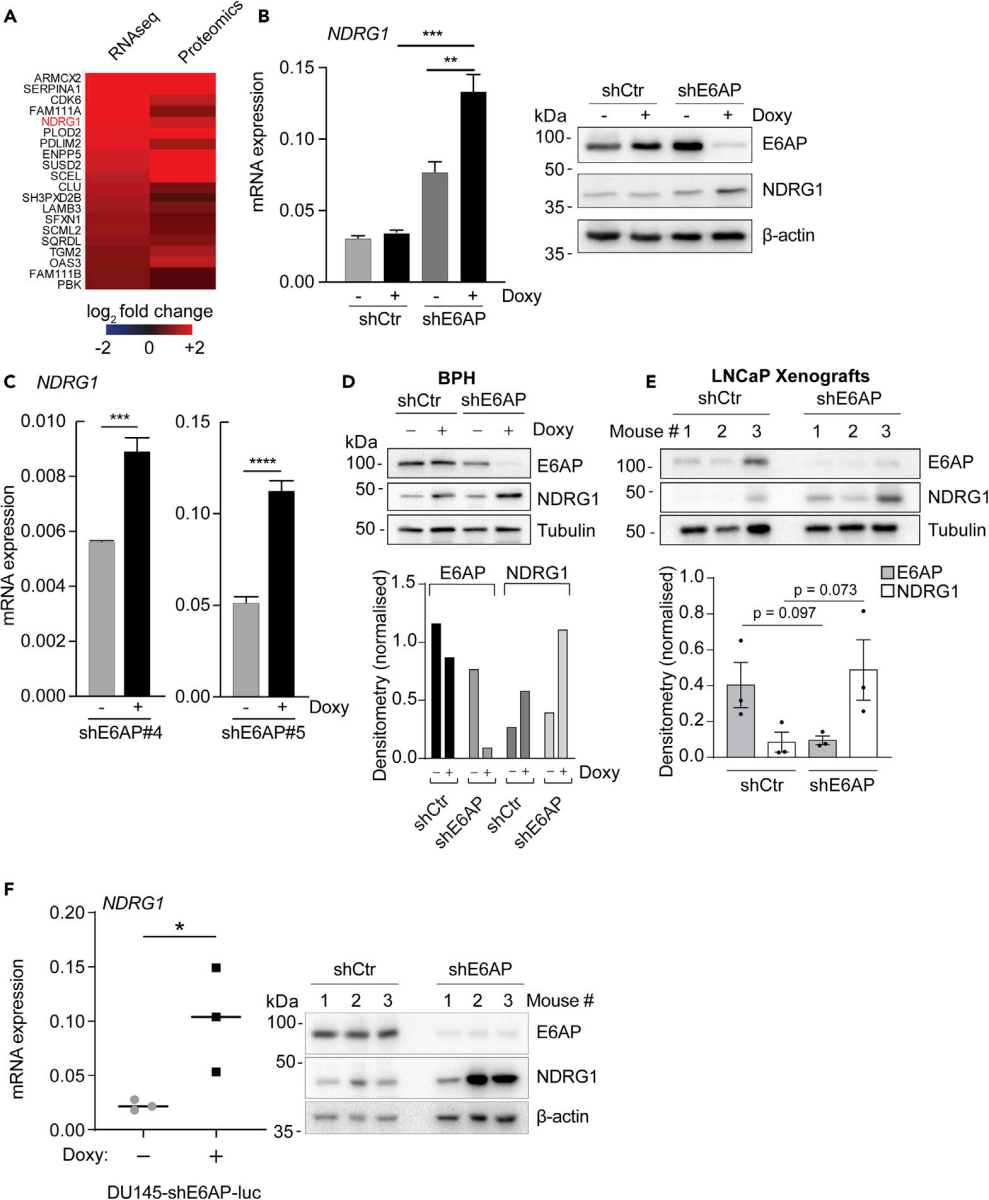
E6AP Represses the Levels of the Metastatic Suppressor NDRG1 (A) Heatmap of the top 20 significantly commonly upregulated transcripts and proteins upon E6AP KD in DU145 cells from the combined transcriptomics and proteomics screen depicted in Figure 2A. (B) RT-PCR (left) and immunoblotting (right) of *NDRG1* mRNA and protein levels in DU145-shE6AP and control cells (shCtr), treated with Doxy for three days or remained untreated. mRNA data are ΔΔC_t_±SD of technical triplicates of one of three independent experiments. Immunoblot is representative of three independent experiments. **p < 0.01, ***p < 0.001, unpaired *t* test. (C) RT-PCR of *NDRG1* mRNA in DU145 transduced with two different shRNA against E6AP than the one used in the screen (shE6AP#4 on the left and shE6AP#5 on the right) treated with Doxy for three days and remained untreated. mRNA data are ΔΔC_t_±SD of technical triplicates of one of two independent experiments. ***p < 0.001, ****p < 0.0001, unpaired *t* test. (D and E) (D) Western blot analysis of E6AP, NDRG1, and Tubulin levels in BPH cells (D) and LNCaP xenograft cells (E) following Doxy-induced shE6AP expression (top panels). Bottom panels show densitometry quantification of results and statistical analysis p values where appropriate (paired t test). (F) Endpoint tumor samples were analyzed for NDRG1 mRNA by RT-PCR (left) and immunoblotting (right). β-actin was used as a loading control in the immunoblots. mRNA data are ΔΔC_t_±SD of three technical replicates of three mice.

To identify the relevant targets of E6AP involved in the promotion of metastasis, we analyzed our recently published combined proteomic and transcriptomic screens (Figure 2A; Gulati et al., 2018). The comparison of the significantly altered transcripts and proteins in DU145 cells upon E6AP KD identified 142 commonly altered candidates (Gulati et al., 2018). Notably, one of the top candidates that was upregulated upon E6AP KD was NDRG1 (Figure 7A). NDRG1 is a *bona fide* metastasis suppressor in PC, frequently repressed in PC patients with high Gleason grade (Chung et al., 2012, Song et al., 2010), particularly in patients with loco-regional or distant metastasis (Bandyopadhyay et al., 2003).

To validate NDRG1 as a target of E6AP, we measured the effect of E6AP on the expression of NDRG1 by qPCR and immunoblot in DU145 cells. We confirmed that NDRG1 expression was induced in these cells as well as other PC cell lines upon E6AP KD at the transcript and protein levels (Figures 7B and S4). This was not an off-target effect of the shRNA used in the screen, because *NDRG1* mRNA expression was increased upon E6AP KD using two independent shE6AP in DU145 (Figure 7C).

To study whether the inverse correlation between E6AP and NDRG1 was restricted to DU145 cell line, we extended the analysis to BPH and the poorly metastatic PC line, LNCaP. E6AP KD resulted in a significant increase in the abundance of *NDRG1* mRNA expression levels in both BPH and LNCaP cell lines (Figure 7D). In addition, we measured the effect of E6AP KD on *NDRG1* mRNA and protein levels in an *in vivo* setting using a DU145 xenograft model from our previous study (Paul et al., 2016). qRT-PCR and immunoblotting of tumors collected at the ethical endpoint revealed that KD of E6AP restored NDRG1 mRNA and protein expression levels, as compared with the control tumors (Figures 7E and 7F). These results strongly support association between downregulation of E6AP and increased levels of NDRG1 mRNA and protein in PC *in vitro* and *in vivo*.

### NDRG1 Acts Downstream of E6AP in Regulating Cell Migration

**Figure 8:**
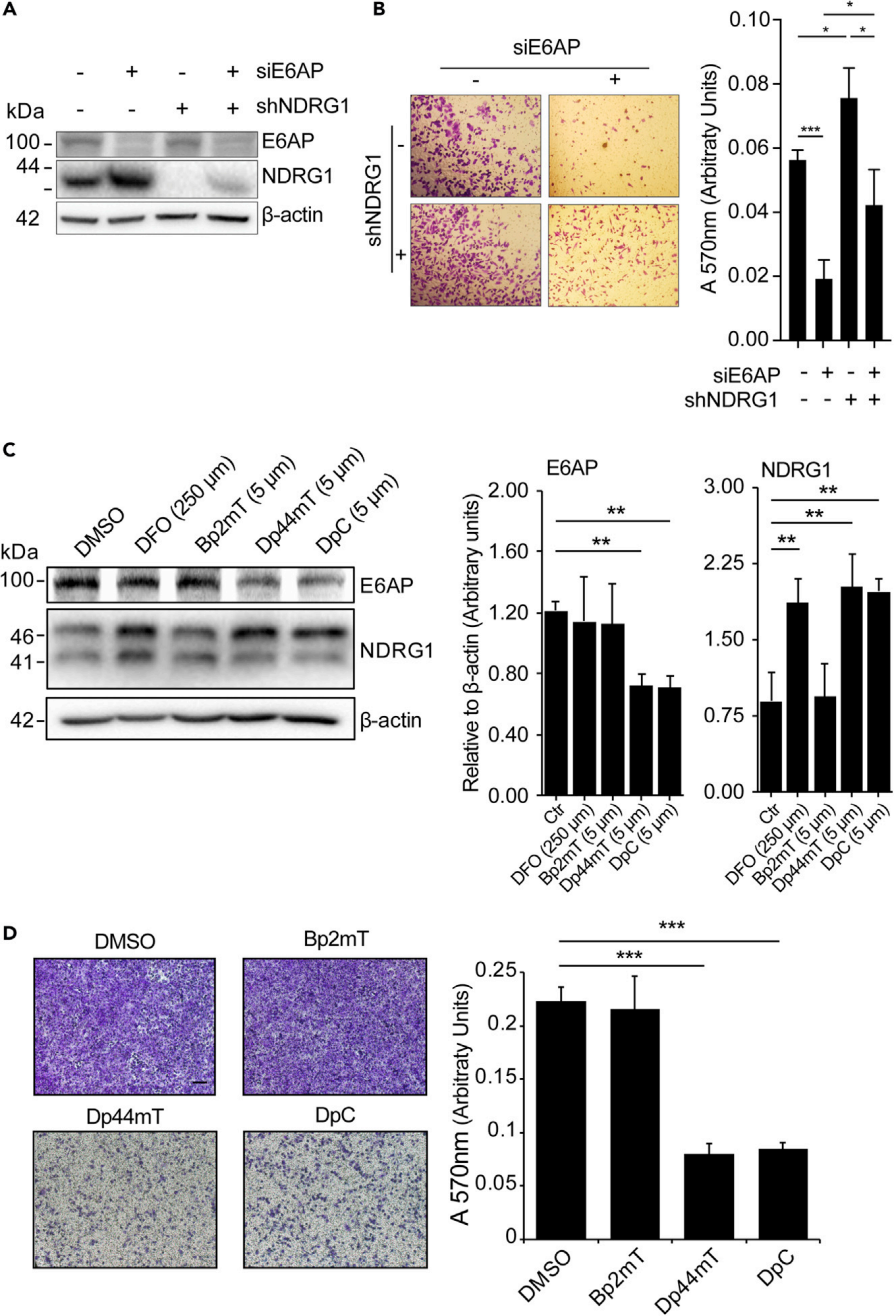
E6AP Promotes Metastatic Phenotype *via* Suppression of NDRG1 that Can Be Restored with Thiosemicarbazones (A) DU145 stably transduced with shNDRG1 (+) or a shRNA control (−) were transiently transfected with a siRNA against E6AP (siE6AP +) or a siRNA control (−). At 72 h post-transfection cells were collected for immunoblot. (B) Cells were seeded in transwells and allowed to migrate for 24 h as described in (A). Invaded cells were stained with crystal violet (left) and quantified at A_570_ after extraction of the dye (right). Each experiment was repeated three independent times. *p < 0.05, ***p < 0.001, unpaired *t* test. (C) DU145 cells were treated with DFO (positive control; 250μm), Bp2mT (negative control analogue; 5μm), Dp44mT, DpC, or Bp2mT (5μm each) for 24 h, and the levels of E6AP and NDRG1 were determined by Western blotting. β-actin was used as a loading control; densitometry quantification of the expression derived from triplicates of one of three independent experiments is shown. **p < 0.01, unpaired *t* test. (D) The effect of the compounds on cell migration was determined by transwell assays. Invaded cells were stained with crystal violet (left) and quantified at A_570_ after extraction of the dye (right). Each experiment was performed in duplicates and repeated three independent times. Quantification of a representative experiment is shown as mean ± SD. Scale bar represents 100 μm.

If NDRG1 is an important downstream target of an E6AP-mediated metastatic phenotype, we would expect that ablation of NDRG1 expression would partially revert the effect of E6AP KD on cell migration. We tested this possibility using DU145 cells stably transduced with a short hairpin against NDRG1 (shNDRG1) or its respective control (shCtr). NDRG1 KD was previously shown to increase cell migration (Sun et al., 2013), consistent with its role as a metastasis suppressor. DU145 cells were transiently transfected with either siRNA specific for E6AP or control siRNA (Figure 8A), and their migratory capacity was measured using the transwell assay. As expected, KD of NDRG1 increased cell migration, whereas E6AP KD reduced it (Figure 8B). Importantly, concomitant downregulation of NDRG1 and E6AP partially restored the effect of shE6AP on cell migration (75% restoration) (Figure 8B). These results support a role for NDRG1 as a key mediator of E6AP effects on cell migration.

### Restoration of NDRG1 Expression Using Thiosemicarbazones

Novel and clinically trialed thiosemicarbazones with potent and marked anti-tumour activity *in vitro* and *in vivo* have been successfully used to increase NDRG1 expression (Chen et al., 2012, Kovacevic et al., 2013, Le and Richardson, 2004, Whitnall et al., 2006, Yuan et al., 2004).

Thiosemicarbazones have been successfully used to restore NDRG1 expression (Yuan et al., 2004). We therefore asked whether this restoration could also be achieved in cells expressing high levels of E6AP. To test this, the thiosemicarbazones, Dp44mT and DpC, were used, and responses were compared with those of the control compound Bp2mT (Stacy et al., 2016), and also a positive control compound, desferrioxamine (DFO; Chen et al., 2012, Whitnall et al., 2006, Yuan et al., 2004).

Treatment of DU145 cells with Dp44mT and DpC elevated NDRG1 levels and reduced E6AP expression (Figure 8C). To measure the effect of the compounds on the migration of DU145 we used a transwell migration assay as described in Figure 3C. DU145 cells were treated with each of the compounds for 24 h prior to the migration assay and during the 24 h migration. Dp44mT and DpC reduced the migration of DU145 by over 60% (Figure 8D). The Bp2mT control compound had no effect on migration (Figure 8D) or on the expression of NDRG1 or E6AP (Figure 8C), indicating that the effects observed in response to Dp44mT and DpC are specific. The effect of these thiosemicarbazone drugs on additional targets cannot be ruled out. Overall, these experiments suggest that thiosemicarbazones can be used to inhibit the migration of PC cells by restoring NDRG1.

## Discussion

E6AP is known to be hijacked by HPV-E6 protein to drive a range of virus-related cancers, including cervical and oral cancers (Beaudenon and Huibregtse, 2008). This effect of E6AP has been largely linked to the degradation of p53, thereby overcoming viral-induced growth inhibition (Beaudenon and Huibregtse, 2008). Over the past decade, E6AP has been also linked to viral-independent cancers, including B-cell lymphoma and lung cancer (Gamell et al., 2017b, Wolyniec et al., 2012). Pertinent to this study, E6AP has been strongly linked to prostate development and PC. Previous studies by others and our laboratory have demonstrated a role for E6AP in the regulation of PC cell growth, survival, and cellular response to multiple stress conditions, including low serum and DNA damage (Khan et al., 2006, Paul et al., 2016, Srinivasan and Nawaz, 2011, Wolyniec et al., 2012). At least three tumor suppressors have been shown to mediate these effects: PML, p27Kip1, and Clusterin (Gulati et al., 2018, Paul et al., 2016, Raghu et al., 2017).

Clinical analyses of PC samples suggested that elevated levels of E6AP are associated with a higher frequency of metastatic cancer development (Birch et al., 2014). These studies prompted us to investigate the role of E6AP in the promotion of metastatic PC directly. Our analysis of clinical samples showed strong association with elevated E6AP expression levels and the formation of regional metastasis (Figure 1). By genetic manipulation of E6AP expression in a variety of PC cell lines, we demonstrated a role for E6AP in the promotion of cell migration, anchorage independent growth (Figures 3 and 5), and in the promotion of lung metastasis of PC cells *in vivo* (Figure 4). This suggests that E6AP enhances either extravasation of cells into the lung and/or early seeding of the lung, as the two cannot be distinguished from this assay.

A crucial process in the promotion of metastasis is the acquisition of a mesenchymal phenotype, including morphological features such as a loss of cell polarity, gain of fibroblastic morphology, loss of cell-cell contact, and growth in a scattered manner (Kalluri and Weinberg, 2009). In a search for the mechanism underlying the impact of E6AP on the metastatic phenotype, we analyzed our combined transcriptomic/proteomic data. This analysis highlighted NDRG1 as the most attractive candidate because it is a known suppressor of metastatic PC. We demonstrated that NDRG1 is a transcriptional target of E6AP in multiple PC cell lines (Figure 7). Importantly, we showed that NDRG1 is a key target in the effect of E6AP on the migration of PC cells (Figure 8), an effect that has also been demonstrated in other cancers including that of the prostate (Chen et al., 2012, Liu et al., 2015, Sun et al., 2013).

Over-expression of NDRG1 in PC cells inhibits their metastatic phenotype (Bandyopadhyay et al., 2003, Sun et al., 2013). This is achieved by regulating multiple signaling pathways, including NF-κB, PI3K, and ERK, thereby preventing epithelial-to-mesenchymal transition, among other metastatic functions (reviewed in Bae et al., 2013). This provides an explanation, at least in part, for the mechanism by which E6AP impacts on the observed mesenchymal phenotype and consequently on metastasis. It supports the link between E6AP and PI3 kinase as previously observed in PC cells (Srinivasan and Nawaz, 2011). The contribution of NDRG1 to the metastatic phenotype of E6AP does not exclude the contribution of other known targets of E6AP potentially, including PML, p27Kip1, and Clusterin, all of which are tumor suppressors whose expression is partially or completely lost in aggressive and metastatic PC, among other cancers (3,4,9).

In addition to the regulation of NDRG1 expression, we demonstrated that E6AP overexpression results in the upregulation of *Slug* and *Snail*, β-catenin, and vimentin, key markers of mesenchymal phenotype and downstream targets of TGFββ (Figures 5 and 6). This opens the exciting possibility that E6AP might promote metastasis by regulating NDRG1 levels and potentiating TGFβ-mediated epithelial-to-mesenchymal transition. Consistent with this hypothesis, downregulation of NDRG1 in PC cells was previously shown to mimic TGFβ-induced mesenchymal switch (Chen et al., 2012).

Metastatic PC is a major health issue due to the lack of durable responses to conventional therapeutic options. Restoration of tumor suppression provides an attractive therapeutic approach. This can be achieved by global inhibition of proteasomal degradation. This approach had some success in clinical trials in PC, and a new generation of proteasome inhibitors is currently in clinical development (reviewed in Yang et al., 2009). However, given the nature of this global approach, it is not unexpected that it is associated with side effects. An alternative approach is to restore the expression and function of specific key tumor suppressors, such as NDRG1. This can be achieved by protecting these tumor suppressors from degradation by specifically targeting their E3 ligases. This is exemplified by the protection of p53 from MDM2-mediated degradation, using nutlin (reviewed in Haupt et al., 2017). Alternatively, the expression of the tumor suppressor may be elevated by small molecules.

Novel second-generation thiosemicarbazone compounds are now available and the most potent of these agents, DpC, is currently in a clinical trial in Australia for patients with advanced solid tumors (NCT02688101). These compounds exert their anti-cancer activity, at least in part, by upregulating NDRG1 (Le and Richardson, 2004, Sun et al., 2013, Whitnall et al., 2006). This has been proposed to occur through the depletion of cellular iron *via* HIF1-α-dependent and -independent pathways (Le and Richardson, 2004). We show that thiosemicarbazones significantly reduce the migration of DU145, at doses that reduce E6AP levels and restore NDRG1 expression (Figure 8). These effects are similar to those achieved by KD of E6AP, including reduction in migration (Figure 3A) and restoration of NDRG1 expression (Figure 7). Crucially, thiosemicarbazones have already been shown to inhibit metastasis *in vivo*, including in osteosarcoma (Li et al., 2016), hepatocellular carcinoma (Wang et al., 2014), and breast cancer (Liu et al., 2012), highlighting their utility in combating metastasis in multiple cancers. In the latter study the effect of Dp44mT was associated with upregulation of NDRG1 expression *in vivo*. We note that the effect of thiosemicarbazones on additional targets in parallel with NDRG1 cannot be ruled out.

We therefore propose that in addition to the known mechanisms by which thiosemicarbazones act, they also reduce migration of PC cells by reducing E6AP levels, which contribute to the overall restoration of NDRG1 expression. These compounds may provide an attractive therapeutic option for the treatment of metastatic PC with elevated levels of E6AP and downregulation of NDRG1.

### Limitations of the Study

• The experiments are based on knockdown of E6AP, without the inclusion of knockout experimental system. Prostate cancer cells do not tolerate depletion of E6AP.
• Thiosemicarbazones affect multiple targets in addition to NDRG1.
• The *in vivo* evidence is based on one experimental model.

## Methods

All methods can be found in the accompanying Transparent Methods supplemental file.

## Supplemental Information

Document S1. Transparent Methods, Figures S1–S5, and Table S1
