# Supplementary material for: E6AP Promotes a Metastatic Phenotype in Prostate Cancer
Source: iScience. 2019 Nov 2;22:1–15. doi: 10.1016/j.isci.2019.10.065 (PMC6864340; doi:10.1016/j.isci.2019.10.065)
Supplement: Document S1. Transparent Methods, Figures S1–S5, and Table S1 [file mmc1.pdf]

ISCI, Volume *22*

## Supplemental Information

# E6AP Promotes a Metastatic Phenotype in Prostate Cancer

**Cristina Gamell, Ivona Bandilovska, Twishi Gulati, Arielle Kogan, Syer Choon Lim, Zaklina Kovacevic, Elena A. Takano, Clelia Timpone, Arjelle D. Agupitan, Cassandra Litchfield, Giovanni Blandino, Lisa G. Horvath, Stephen B. Fox, Scott G. Williams, Andrea Russo, Enzo Gallo, Piotr J. Paul, Catherine Mitchell, Shahneen Sandhu, Simon P. Keam, Sue Haupt, Des R. Richardson, and Ygal Haupt**

## Supplementary Figures

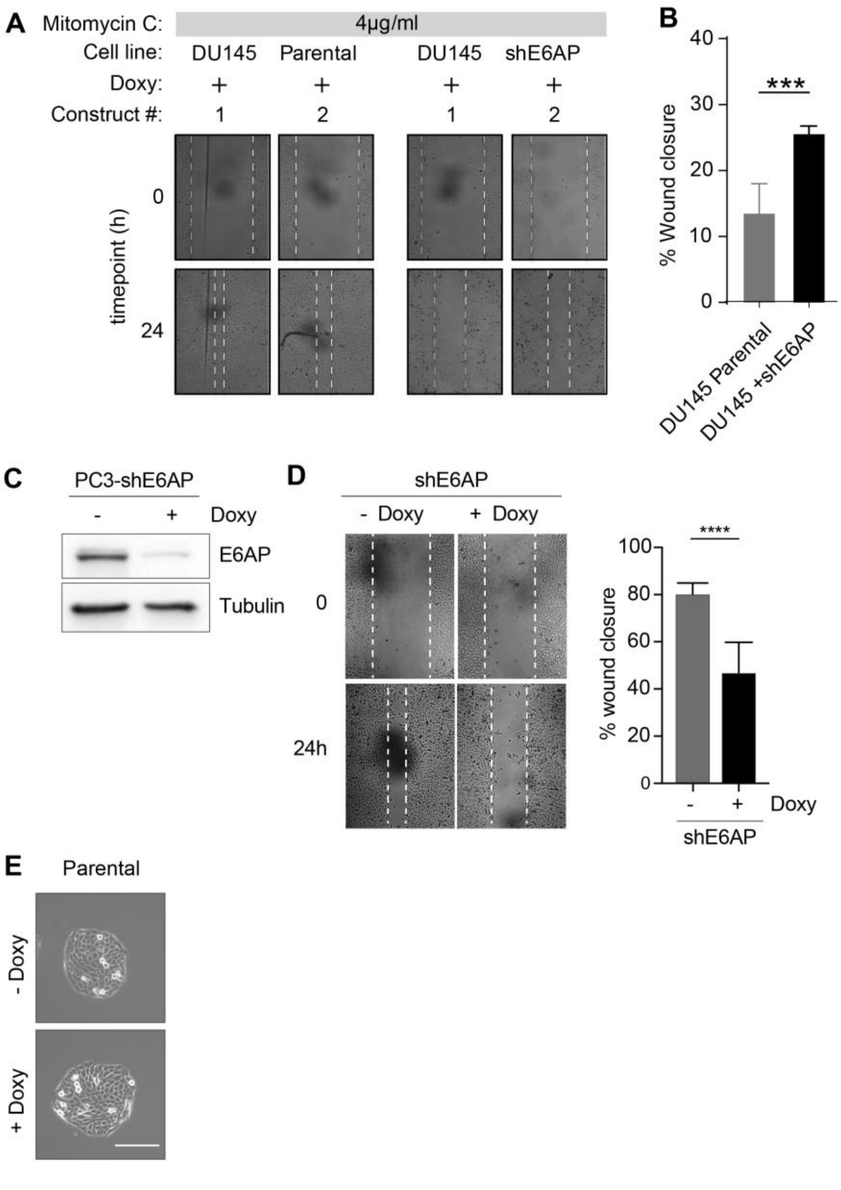


**Figure S1: E6AP knockdown reduces the migration capacity of PC cell line PC3. Related to Figure 3. (A)** Migration of DU145 parental and shE6AP-expressing cells in the presence of Doxycycline (Doxy) and 4μg/mL Mitomycin C. **(B)** Quantification of wound closure from (A). **(C)** Immunoblot confirming knockdown (KD) of E6AP upon Doxy treatment for 3 days in PC3-shE6AP cells. **(D)** PC3-shE6AP cells were treated with Doxy for 3 days before the confluent cell monolayer was scratched. Cells were then allowed to migrate in the presence or absence of Doxy for 24h. Representative images are shown on the left. Quantitative analysis of wound closure was obtained from multiple photographed fields immediately following the scratch, and at end-point (right panels). Each experiment was performed in duplicates and repeated two independent times. Quantification of a representative experiment is shown as mean ± SD.

****$p<0.0001$, unpaired *t*-test. (E) Morphology of parental DU145 cells grown in presence of Doxy. Scale bar: 100µm.

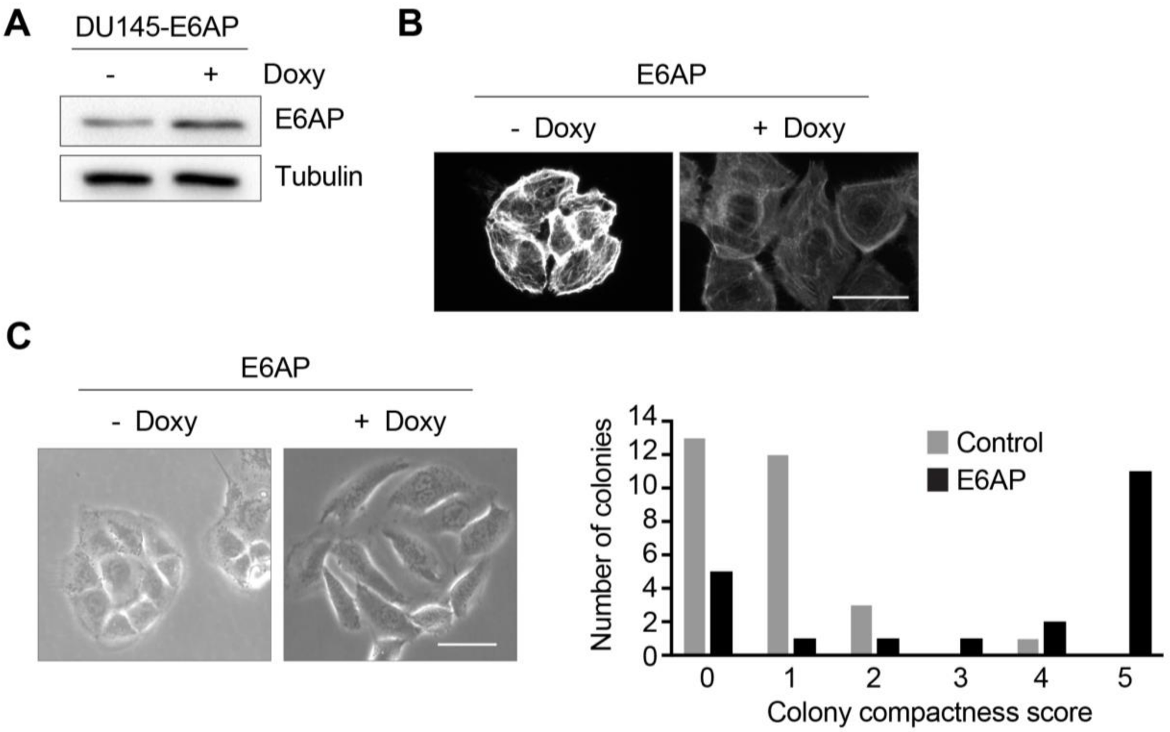


**Figure S2: E6AP overexpression in DU145 cells promotes their metastatic potential. Related to Figure 5 (A)** Immunoblot confirming overexpression of E6AP upon Doxy treatment for 3 days in DU145-E6AP cells. **(B)** DU145-E6AP cells were cultured for 3 days in the presence or absence of Doxy. Actin was then visualized with rhodamine-conjugated phalloidin using a fluorescent microscope. Scale bar: 50µm. Each experiment was performed in at least triplicates and repeated 3 independent times. **(C)** DU145-E6AP cells were plated at low density and allowed to form colonies for 5 days in the presence or absence of Doxy. Representative phase-contrast images of the colonies formed are shown. Scale bar: 50µm. Each experiment was performed in triplicates and repeated three independent times.

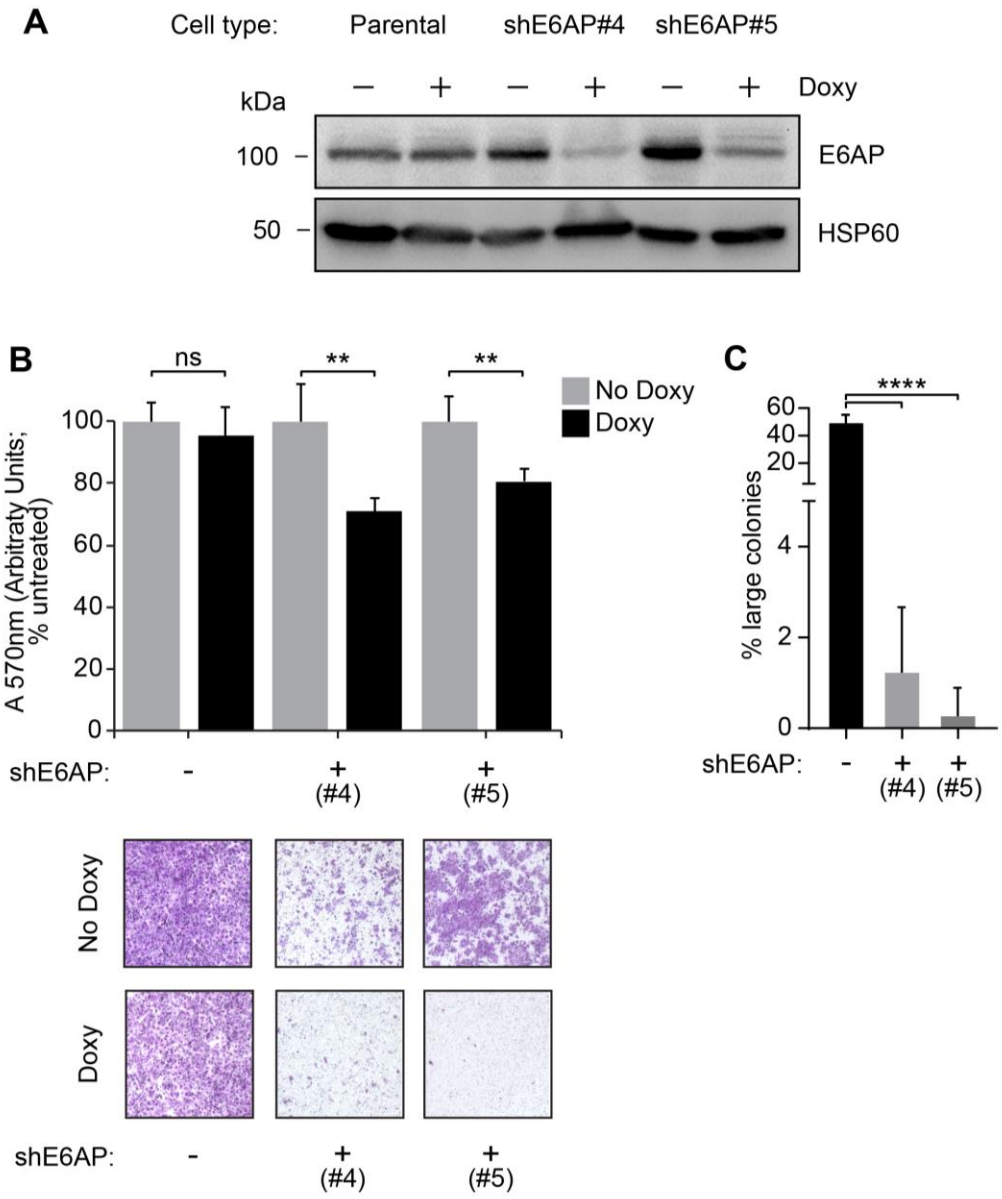


**Figure S3: E6AP knockdown with 2 alternative short hairpins RNAs against E6AP reduces the metastatic potential of PC cells *in vitro*. Related to Figure 3,**
**(A)** Immunoblot confirming the KD of E6AP upon Doxy treatment (200ng/mL) for 3 days in DU145 shE6AP #4 and #5. **(B)** DU145-shE6AP #4 and #5 cells were treated with Doxy for 2.5 days, seeded in transwells and allowed to migrate in the presence or absence of Doxy for 24h. Invaded cells were stained with crystal violet (bottom) and quantified at $A_{570}$ after extraction of the dye (top). Quantification of a representative experiment is shown as mean ± SD. **(C)** DU145-shE6AP cells were grown in soft agar in the presence or absence of Doxy for 11 days as described in Fig. 3D. Colonies sizes were quantified as

in Fig. 3. Large-size colony percentages were calculated as a proportion of total cells and/or colonies in each magnification field. ****$p$<0.0001, unpaired $t$-test.

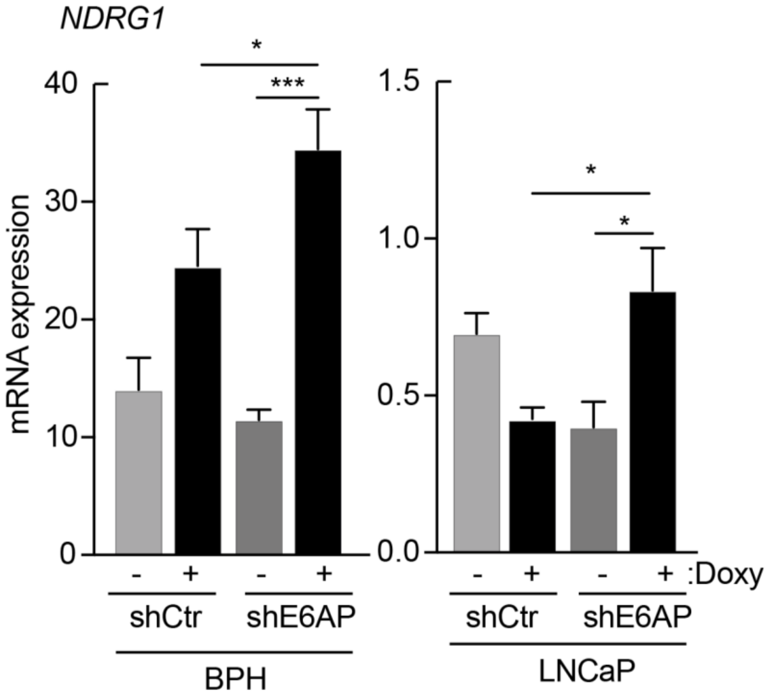


**Figure S4: Quantification of NDRG1 mRNA changes in cells with E6AP knockdown. Related to Figure 7.** RT-PCR of *NDRG1* mRNA in BPH (left) and LNCaP (right) transduced with the shRNA against E6AP used in the original screen treated with Doxy for 3 days. mRNA data is $\Delta\Delta C_t \pm SD$ of technical triplicates of one of two independent experiments. *$p$<0.05, ***$p$<0.001, unpaired $t$-test.

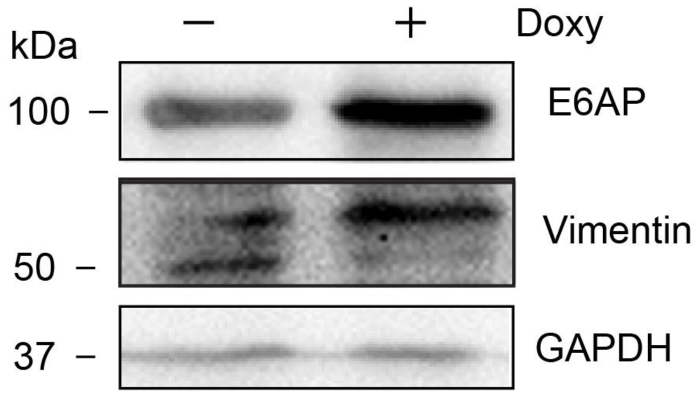


**Figure S5. Overexpression of E6AP in BPH cells increases expression of mesenchymal marker Vimentin. Related to Figure 5.** BPH-E6AP cells were exposed to Doxy (200ng/mL) for 9 days. Protein expression of Vimentin and E6AP was determined by immunoblot. Expression of GAPDH was used as loading control.

**Table S1. List of primers used. Related to Figure 5 and Figure 6.**

| Gene name | Forward primer (5’-3’) | Reverse primer (5’-3’) |
|---|---|---|
| *E6AP* | GAGGCATTGGTACAGAGCTTC | CCTGTGAGCTATCACCTATCCT T |
| *RPL37a (internal control)* | GCC AGC ACG CCA AGT ACA C | CCC CAC AGC TCG TCT CTT CA |
| *SNAI2 (Slug)* | CGAACTGGACACACATACAGTG | CTGAGGATCTCTGGTTGTGGT |
| *SNAI1 (Snail)* | TCGGAAGCCTAACTACAGCGA | AGATGAGCATTGGCAGCGAG |
| *NDRG1* | CCAACAAAGACCACTCTCCTC | CCATGCCCTGCACGAAGTA |

## Transparent Methods

**Cell culture and reagents.** All cell lines were obtained from the American Type Culture Collection. DU145 and PC3 were maintained in DMEM. LNCaP and BPH were cultured in RPMI. All were supplemented with 10% foetal bovine serum (FBS) and 0.1% penicillin/streptomycin, and cells were cultured at 37˚C. The reagents used in cellular experiments were Mitomycin C (Sigma) and recombinant human TGFβ (PreProtech). The thiosemicarbazones, Dp44mT and DpC, and the respective negative control, Bp2mT, were synthesized and characterized as previously described (Lovejoy et al., 2012; Richardson et al., 2006; Stacy et al., 2016), while desferrioxamine (DFO) was purchased from Sigma-Aldrich (St. Louis, MO). Dp44mT, DpC and Bp2mT were dissolved in dimethyl sulfoxide (DMSO) and further diluted to final concentration of 5µm in culture media, whereas DFO was diluted in culture media to final concentration of 250µm. Cells were incubated with either DFO, Bp2mT, Dp44mT or DpC for 24h prior to protein extraction and Western analysis.

**Proteomics and Transcriptomics.** Details for proteomic and transcriptomic analysis of E6AP KD in DU145 cells, including deposition of raw data, are provided in previous study (Gulati et al., 2018).

**Data and Software Availability.** Raw data for proteomics and transcriptomics is deposited at ProteomeXchange (PXD008743) and Gene expression omnibus (GSE107245), respectively.

**Plasmids and lentivirus generation.** The sequences for lentiviruses expressing shRNA against E6AP (shE6AP) and its control (shControl) and methodology for viral production and infection have previously been described (Paul et al., 2016). To overexpress E6AP, the human E6AP full sequence was cloned into FUV1-GFP lentiviral vector. The vector control FUV1 was used as a control. KD or overexpression of E6AP was induced with 0.2µg/mL Doxycycline (Doxy; Sigma-Aldrich) in DU145 and PC3, 0.1µg/mL Doxy in BPH cells and 0.05µg/mL Doxy in LNCaP. To KD E6AP with the siRNA, the siGENOME SMARTpool

siRNA against E6AP was purchased from Dharmacon (M-005137-00-0005). A scrambled control siRNA was transfected under the same conditions. DU145 cells that stably express a short hairpin RNA against NDRG1 were previously generated (Chen et al., 2012).

**Prostate cancer TMA staining and analysis.** All studies carried out on human specimens were approved by the Peter MacCallum Cancer Centre Human Ethics Committee. The Rome cohort TMA contained PC biopsies collected from the Urology Department at IRCCS Regina Elena National Cancer Institute, Rome, Italy. The biopsies used were the archived samples of patients who underwent radical prostatectomy without any pharmacological treatment. Samples were analysed and stained for E6AP using anti-E6AP (1:400 dilution, MCA3532Z, AbD Serotec). IHC slides were semi-quantitatively scored. High E6AP was defined as maximum nuclear expression across the core ≥80% staining versus low E6AP maximum nuclear expression defined as <80% (Birch et al., 2014). Each prostatectomy specimen was represented by a 1-mm-diameter tumour core. The specimens were assessed by a specialist histopathologist who was blinded to patient outcome. Nuclear expression of E6AP was scored as the percentage of cancer cells stained.

**Bioinformatics and statistical analysis.** Results are reported as mean ± standard deviation (SD) or mean ± standard error of the mean (SEM), as indicated in figure legends. Statistical significance was determined by two-sided Student's *t*-test. For all statistical comparisons, $p < 0.05$ was considered significant. The gene ontology (GO) and pathway analysis of the shortlisted transcripts and proteins from the omics screens was performed on Enrichr, a dataset within the ENCODE project (Chen et al., 2013; Kuleshov et al., 2016). GO enrichment for cellular component 2018 and biological pathways generated using Reactome 2016 were subjected to Fisher's exact test (*p*-value < 0.05) and z-score computation to generate the combined score. Gene set enrichment analysis (GSEA) was conducted using GSEA v3.0 on various functional and/or characteristic signatures (Subramanian et al., 2005). Gene sets were obtained from MSigDB database v6.2. GSEA results are shown using the normalised enrichment score (NES),

which accounts for differences in gene set size and correlation between genes in different datasets, and FDR.

**Tail vein injection assay of cancer metastasis.** To evaluate lung metastatic capacity, DU145-shE6AP-Luc cells were treated with Doxy (200ng/mL) for 2.5 days and injected ($1x10^6$/100µl suspension) in the tail vein of 6-8 weeks old NSG (*n*=6 per group) that had been previously injected intraperitoneally with Doxy (2 mg/kg) and provided Doxy in drinking water (2mg/mL). Control mice were not exposed to Doxy. Lung metastasis formation was monitored by *in vivo* bioluminescence. Briefly, D-Luciferin (Xenogen) was injected intraperitoneally at 150mg/kg and mice were allowed to move freely to aid in distribution of luciferin. After 5 min mice were anaesthetised using isofluorane before transfer into the imaging chamber of a Lumina II instrument and placed into nose cones. Using IVIS Living Image 3.0 software, images were taken. Mice were typically imaged weekly and weighed twice weekly. Image acquisition and processing was accomplished using the Living Image software (PerkinElmer). Mice were culled when breathing difficulties became evident in line with ethical approval. Autopsies were performed immediately to evaluate for the presence of metastatic tumours.

**Wound healing assay.** Cells were plated in 96 well plates and grown to a confluent monolayer for 3 days in the presence or absence of Doxy. The 'scratch' was introduced by scraping the cell monolayer using a pin tool (Custom FP3-WP, Flat tip, Parlene coated, 1.67mm diameter, mounted on BMPZYMARK mounting plate from V&P Scientific, Inc.) on a Sciclone ALH3000 Workstation robot (Caliper Life Sciences, Hopkinton, MA). Cells were then washed with medium to remove detached cells and photographed with an inverted Zeiss Axiovert microscope immediately and 24h later. The rate of migration was measured as the percentage of invaded area with respect to the initial wound area using ImageJ.

**Transwell assay.** Chemotaxis assays were performed in 24-well Transwell plates using 8µm pore-size polycarbonate filters of 8 mm diameter. Cells pre-treated with Doxy for 3 days were subsequently

trypsinised and a suspension of 100µl containing $1\times10^6$ cells in media with 0.05% FBS was loaded onto the upper chamber. The lower chamber was filled with media with 20% FBS. After 24h, non-migrated cells in the top chamber were removed with a cotton swab and migrated cells were fixed and stained with a solution containing 0.4% crystal violet and 20% methanol for 20 min. The transwells were thoroughly washed with water to remove excess dye and subsequently dried. Images were acquired using an inverted Zeiss Axiovert microscope. Cell migration was quantified by eluting the crystal violet dye by incubating the transwell with 33% acetic acid 5 min in agitation and reading its absorbance with a spectrophotometer plate reader at 570 nm.

**Soft agar assay.** Growth in anchorage-independent condition was assessed by colony formation in low melting agarose. Cells were resuspended at a density of $2.5\times10^3$ in 0.375% agarose (Invitrogen) in complete media with Doxy and seeded into 12 well plates coated with 0.75% agarose. The plates were incubated at 37°C for 11 days. Colonies were visualized using the inverted Zeiss Axiovert microscope with cell sizes enumerated using Fuji Imaging Software according to thresholds provided in the figure legends.

**Immunoblotting.** Cells were lysed with 50mM Tris-HCl pH 7.4, 250mM NaCl, 5mM EDTA, 0.1% Triton X-100 supplemented with proteases and phosphatases inhibitors. Western blots were proven with antibodies against E6AP (E8655, Sigma), β-Tubulin (T2200, Sigma), NDRG1 (ab37897, Abcam), Vimentin (3932, Cell Signaling), β-catenin (610153, BD Bioscience) and GAPDH (ab9484, Abcam).

**Phalloidin staining.** Cells were grown on glass coverslips in 12-well plates in the presence or absence of Doxy for 3- or 4-days as detailed in the figure legends. Cells were fixed in 4% paraformaldehyde in PBS for 30 min at room temperature, washed twice in PBS and permeabilized for 10mins in PBS containing 0.1% Triton X-100, and then blocked in TBS containing 5% BSA for 30mins. To visualize F-actin, cells were incubated with 1:100 Rhodamine-conjugated phalloidin (ThermoFisher) and washed three times

with PBS before mounting on slides. Images were acquired using a BX-51 fluorescent microscope. For some experiments, 2 days after Doxy treatment, cells were starved in serum-free medium for 16 hours and then stimulated with 10ng/mL ♀TGFβ (PeproTech) for 48h in the presence of Doxy.

**Cell colony scattering assay.** $1x10^3$ cells were plated in 6-well plates in the presence or absence of Doxy for 4 or 5 days as detailed in the figure legends. For some experiments, 2 days after Doxy treatment, cells were starved in serum-free medium for 16h and then stimulated with 10ng/mL TGFβ (PeproTech) for 48h in the presence of Doxy.

**RNA extraction and qPCR.** RNA was isolated using TriZol Reagent (Life Technologies) in accordance with the manufacturer's instructions. cDNA was synthesized using M-MLV reverse transcription kit (Promega) and Random Primers (Promega). PCR was performed on StepOnePlus PCR machine (Applied Biosystems) using Fast SYBR Green Master Mix (Applied Biosystems). The primer sequences are detailed in Table S1.

## Supplemental References


Birch, S. E., Kench, J. G., Takano, E., Chan, P., Chan, A. L., Chiam, K., Veillard, A. S., Stricker, P., Haupt, S., Haupt, Y., *et al.* (2014). Expression of E6AP and PML predicts for prostate cancer progression and cancer-specific death. Annals of oncology : official journal of the European Society for Medical Oncology / ESMO *25*, 2392-2397.

Chen, E. Y., Tan, C. M., Kou, Y., Duan, Q., Wang, Z., Meirelles, G. V., Clark, N. R., and Ma'ayan, A. (2013). Enrichr: interactive and collaborative HTML5 gene list enrichment analysis tool. BMC bioinformatics *14*, 128.

Chen, Z., Zhang, D., Yue, F., Zheng, M., Kovacevic, Z., and Richardson, D. R. (2012). The iron chelators Dp44mT and DFO inhibit TGF-beta-induced epithelial-mesenchymal transition via up-regulation of N-Myc downstream-regulated gene 1 (NDRG1). J Biol Chem *287*, 17016-17028.

Gulati, T., Huang, C., Caramia, F., Raghu, D., Paul, P. J., Goode, R. J. A., Keam, S. P., Williams, S. G., Haupt, S., Kleifeld, O., *et al.* (2018). Proteotranscriptomic Measurements of E6-Associated Protein (E6AP) Targets in DU145 Prostate Cancer Cells. Molecular & cellular proteomics : MCP *17*, 1170-1183.

Kuleshov, M. V., Jones, M. R., Rouillard, A. D., Fernandez, N. F., Duan, Q., Wang, Z., Koplev, S., Jenkins, S. L., Jagodnik, K. M., Lachmann, A., *et al.* (2016). Enrichr: a comprehensive gene set enrichment analysis web server 2016 update. Nucleic Acids Res *44*, W90-97.

Lovejoy, D. B., Sharp, D. M., Seebacher, N., Obeidy, P., Prichard, T., Stefani, C., Basha, M. T., Sharpe, P. C., Jansson, P. J., Kalinowski, D. S., *et al.* (2012). Novel second-generation di-2-pyridylketone thiosemicarbazones show synergism with standard chemotherapeutics and demonstrate potent activity against lung cancer xenografts after oral and intravenous administration in vivo. J Med Chem *55*, 7230-7244.

Paul, P. J., Raghu, D., Chan, A. L., Gulati, T., Lambeth, L., Takano, E., Herold, M. J., Hagekyriakou, J., Vessella, R. L., Fedele, C., *et al.* (2016). Restoration of tumor suppression in prostate cancer by targeting the E3 ligase E6AP. Oncogene *35*, 6235-6245.

Richardson, D. R., Sharpe, P. C., Lovejoy, D. B., Senaratne, D., Kalinowski, D. S., Islam, M., and Bernhardt, P. V. (2006). Dipyridyl thiosemicarbazone chelators with potent and selective antitumor activity form iron complexes with redox activity. J Med Chem *49*, 6510-6521.

Stacy, A. E., Palanimuthu, D., Bernhardt, P. V., Kalinowski, D. S., Jansson, P. J., and Richardson, D. R. (2016). Structure-Activity Relationships of Di-2-pyridylketone, 2-Benzoylpyridine, and 2-Acetylpyridine Thiosemicarbazones for Overcoming Pgp-Mediated Drug Resistance. J Med Chem *59*, 8601-8620.

Subramanian, A., Tamayo, P., Mootha, V. K., Mukherjee, S., Ebert, B. L., Gillette, M. A., Paulovich, A., Pomeroy, S. L., Golub, T. R., Lander, E. S., and Mesirov, J. P. (2005). Gene set enrichment analysis: a

knowledge-based approach for interpreting genome-wide expression profiles. Proc Natl Acad Sci U S A *102*, 15545-15550.
